# Context-Aware Alerting in Elderly Care Facilities: A Hybrid Framework Integrating LLM Reasoning with Rule-Based Logic

**DOI:** 10.3390/s25216560

**Published:** 2025-10-24

**Authors:** Nazmun Nahid, Md Atiqur Rahman Ahad, Sozo Inoue

**Affiliations:** 1 Graduate School of Life Science and Systems Engineering, Kyushu Institute of Technology, 2-4 Hibikino, Wakamatsu Ward, Kitakyushu 808-0135, Japan; sozo@brain.kyutech.ac.jp; 2 Department of Engineering & Computing, School of Architecture Computing and Engineering, University of East London, London E16 2RD, UK

**Keywords:** alarm fatigue, large language model, fall detection, context-aware systems, nurse alerting, long-term care, nurse care

## Abstract

The rising demand for elderly care amid ongoing nursing shortages has highlighted the limitations of conventional alert systems, which frequently generate excessive alerts and contribute to alarm fatigue. The objective of this study is to develop a hybrid, context-aware nurse alerting framework for long-term care (LTC) facilities that minimizes redundant alarms, reduces alarm fatigue, and enhances patient safety and caregiving balance during multi-person care scenarios such as mealtimes. To do so, we aimed to intelligently suppress, delay, and validate alerts by integrating rule-based logic with Large Language Model (LLM)-driven semantic reasoning. We conducted an experimental study in a real-world LTC environment involving 28 elderly residents (6 high, 8 medium, and 14 low care levels) and four nurses across three rooms over seven days. The proposed system utilizes video-derived skeletal motion, care-level annotations, and dynamic nurse–elderly proximity for decision making. Statistical analyses were performed using F1 score, accuracy, false positive rate (FPR), and false negative rate (FNR) to evaluate performance improvements. Compared to the baseline where all nurses were notified (100% alarm load), the proposed method reduced average alarm load to 27.5%, achieving a 72.5% reduction, with suppression rates reaching 100% in some rooms for some nurses. Performance metrics further validate the system’s effectiveness: the macro F1 score improved from 0.18 (baseline) to 0.97, while accuracy rose from 0.21 (baseline) to 0.98. Compared to the baseline error rates (FPR 0.20, FNR 0.79), the proposed method achieved drastically lower values (FPR 0.005, FNR 0.023). Across both spatial (room-level) and temporal (day-level) validations, the proposed approach consistently outperformed baseline and purely rule-based methods. These findings demonstrate that the proposed approach effectively minimizes false alarms while maintaining strong operational efficiency. By integrating rule-based mechanisms with LLM-based contextual reasoning, the framework significantly enhances alert accuracy, mitigates alarm fatigue, and promotes safer, more sustainable, and human-centered care practices, making it suitable for practical deployment within real-world long-term care environments.

## 1. Introduction

With aging, the human body undergoes inevitable physiological and cognitive changes. Elderly individuals experience mobility impairment, chronic illnesses, and cognitive decline. This makes them more prone to injuries and falls. Among the causes of injury, fall incidents are prominent as a major public health concern. Each year, nearly 36 million older adults experience a fall, with 3 million requiring emergency care [1]. Falls often result in severe outcomes, including fractures (64%), post-fall trauma or fear (44%), and hospitalizations (32%) [2,3]. These episodes can also lead to long-term consequences, such as reduced autonomy, immobility, dependence, depression, and an overall decline in functional capacity. Despite increasing awareness, the effectiveness of traditional fall prevention strategies—such as scheduled check-ins or individualized physical training—has been limited [4,5]. For healthcare personnel, the need to continuously monitor vulnerable elderly patients adds substantial physical and cognitive workload, especially in long-term care (LTC) environments where nurse-to-patient ratios are often stretched. Consequently, the development of proactive and intelligent fall detection and alert systems has garnered significant interest across both clinical and scientific domains.

With the advancement of information and communication technologies, a variety of smart healthcare solutions have emerged to support fall detection and early warning systems [6,7,8,9]. While such systems showed promising results [8,10,11], the existing alert systems often interrupt clinical workflow [12,13], and may compromise safety outcomes [14,15,16]. The reason for safety concern is that the alerts often overwhelm caregivers. This phenomenon, widely known as ‘alarm fatigue’, is now recognized as a critical threat to patient safety and caregiver well-being [17,18]. Alarm fatigue occurs when healthcare workers are exposed to a large volume of alerts—most of which are either irrelevant, false, or not actionable [18]. These interruptions fragment caregiver workflow, elevate stress levels, and introduce a high cognitive burden that diminishes the quality of attention paid to actual patient care [19,20]. As a result, caregivers may become desensitized, delay response, or entirely ignore alerts, even when they are valid [21,22].

The main reason behind this situation lies in the pattern that the traditional monitoring systems follows. Most of the systems to ensure patients’ safety tend to send alerts to all the nurses. This leads to an over- and unnecessary alert situation. Moreover, these alert systems lack consideration of the care context to convey the priority of the situation. As health professionals face increasing demands for multitasking and constant responsiveness, the cognitive load caused by alarm over-saturation has become a crisis. False alarms and poorly prioritized alerts fragment clinical workflows, reduce trust in alert systems, and contribute to staff burnout and techno-stress. Many nurses report difficulty discerning alert priority, locating the source of alarms, and responding effectively due to environmental noise or non-clinical disruptions [23]. The problems that arise due to poor clinical alarm management can be categorized mainly in three categories [24]:

- False Alarms: False alarms can be both positive and negative. A false positive alarm occurs when an alert is triggered even though there is no actual clinical need or emergency. A false negative alarm refers to a situation where a clinically important event occurs, but the system fails to detect or alert it.
- Unreachable Alarms: An unreachable alarm refers to an alert that is sent out but fails to reach the appropriate caregiver in time. This can be due to routing inefficiencies where an alert is sent to a nurse at such a time when the time to reach the elderly patient is not enough.
- Unattainable Alarms: An unattainable alarm is triggered and sent to a caregiver who is occupied or too far away to respond at the moment the event occurs.

**Figure 1:**
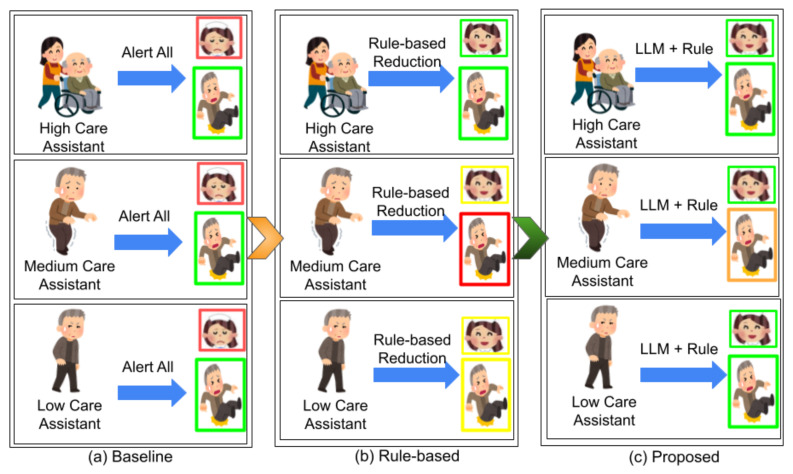
Illustration demonstrating the need for a context-aware alarm system. Panel (**a**) represents the baseline scenario where all nurses receive alerts, (**b**) depicts reduced alerts through rule-based logic, and (**c**) shows further refinement when LLM-based validation is integrated with rule-based methods. For nurses, ‘red’ indicates overload from frequent alarms, ‘yellow’ reflects a moderate reduction in alarm load, and ‘green’ signifies minimal alerts received only when necessary. For elderly residents, ‘red’ denotes a high fall risk, ‘yellow’ indicates a reduced fall risk, ‘orange’ suggests an almost negligible fall risk, and ‘green’ represents no fall risk. Care assistance levels are defined as ‘high’ for residents with care levels 4–5, ‘medium’ for care level 3, and ‘low’ for care levels 1–2.

These factors severely limit the clinical utility of existing alert systems and highlight a critical need for intelligent, context-aware frameworks that issue only the most relevant alerts to the right caregiver, at the right time, to reduce alarm fatigue but ensure care quality, as shown in Figure 1. In the figure, the baseline method is the traditional notify-all nurses approach; the rule-based method includes both rule-based suppression and delay and rule-based suppression, delay, and heuristic validation; and the proposed hybrid model integrates rule-based logic with LLM-driven semantic reasoning.

To address these challenges, this paper presents a feasibility study toward a context-aware nurse alerting system for elderly monitoring in long-term care settings. The proposed system leverages video-derived skeletal data to detect elderly motion, estimate nurse proximity, and intelligently route alerts based on real-time activity analysis and contextual prioritization. Instead of relying solely on rule-based binary thresholds, the system incorporates motion features, estimated distance between the resident and available caregivers, and behavioral markers to dynamically assess risk with the help of an LLM. This approach enables a multi-layered alerting strategy: when a potentially risky activity—such as a resident attempting to stand up unassisted—is detected, an alert is issued to the most suitable nurse based on spatial–temporal metrics; then, a forecast of the resident’s future skeletal motion is generated to assess the likelihood of instability or fall and also analyze nurse responsiveness prompting a second alert if needed. In this study, we addressed three key questions to develop a context-aware nursing alert system:

- Research question 1 (R1): How well does a hybrid alert framework consisting of rule-based logic and LLM reasoning minimize alert fatigue without compromising care quality?
  Contribution: This study introduces a hybrid alerting framework that combines rule-based logic with LLM reasoning to deliver context-aware alerts, aiming to reduce alarm fatigue without compromising care quality. Compared to the baseline scenario where all nurses were alerted (100% alarm load), the proposed framework lowered the average load to 27.5% but achieved an accuracy of 0.98 and a macro F1 score of 0.97, significantly surpassing the baseline (accuracy 0.21, macro F1 score 0.18).
- Research question 2 (R2): To what extent can the inclusion of clinically interpretable information help minimize false alarms in long-term care settings?
  Contribution: Our method leverages clinically interpretable parameters like resident care condition, nurse–resident distance, and mobility to generate alerts, minimizing false alarms. Compared to the baseline (FPR 0.20, FNR 0.79), the method achieves markedly lower error rates (FPR 0.005, FNR 0.023).
- Research question 3 (R3): Is a flexible, context-driven alert validation strategy more effective than static rule-based thresholds in addressing the unique needs of elderly care?
  Contribution: We introduce an LLM-based validation module that incorporates spatial, temporal, and clinical context, instead of rule-based thresholding. Our model achieved an accuracy of 0.98 and a macro F1 score of 0.97, outperforming the rule-based approach (accuracy 0.78, macro F1 score 0.79).

This study advances current elderly monitoring research by introducing a hybrid, context-aware nurse alerting framework that combines rule-based suppression and delay logic with LLM-driven contextual reasoning. Unlike existing systems that rely solely on fixed thresholds or opaque learning models, our approach balances interpretability and adaptability, enabling reliable alert decisions in real-world LTC settings. By integrating spatial (nurse–resident distance), temporal (motion trajectory), and clinical (care level and mobility) information, the framework ensures alerts are both clinically relevant and context-sensitive. Quantitative results demonstrate clear improvements over the baseline and rule-based methods. In alignment with the study’s objectives, the system (1) reduces alarm fatigue through intelligent suppression, (2) enhances alert precision via LLM-based validation, (3) maintains interpretability, and (4) supports balanced, patient-centered caregiving. Overall, this work contributes a scalable, human-centered AI approach that improves safety and efficiency in LTC environments.

## 2. Related Works

Alarm management in healthcare has been a persistent challenge in long-term care (LTC) settings. According to Imhoff et al. [25], the quality of medical device alarms is unsatisfactory, affecting quality of care and patient safety. Numerous strategies have been explored to improve the clinical validity, relevance, and responsiveness of alarm systems. The proposed approaches can be broadly categorized into three categories. In this section, we will elaborately discuss these categories.

### 2.1. Alarm Suppression Algorithms

Alarm suppression algorithms aim to reduce false or clinically non-actionable alerts by enhancing the quality of alarm-generating logic. Several machine learning and statistical approaches have been proposed to address this. Several machine learning methods have been applied for alarm classification and suppression. Random forests and support vector machines have been used to distinguish between true and false alarms, with sensitivity control through voting thresholds in complex physiological time series data [26]. Neural networks, though promising, often face challenges such as long training times, instability in learning for dynamic patient states, and interpretability issues [27]. Fuzzy logic systems are applied in cases where alarm thresholds are not precisely defined, enabling nuanced alert generation [20]. Statistical techniques like autoregressive models, Kalman filters, and spectral regression have been used to model physiological time series for anomaly detection [28]. Time-series-based segmentation and median filtering are used to smooth signals and reduce transient spikes that may trigger false alarms [29]. Autoregressive modeling and Kalman filtering have also been effective in early studies for trend detection and change-point analysis [30]. Feature engineering and dimensionality reduction, such as principal component analysis (PCA) and graphical models, have been adopted to identify the most informative features from high-dimensional physiological data [31]. A lack of clinical interpretability and high false negative risk due to aggressive suppression make these approaches inefficient to deploy in LTC facilities. In summary, while suppression algorithms reduce non-actionable alarms, their limited interpretability and risk of missing critical events make us look for a better context-aware approach [32].

### 2.2. Middleware Solutions and Notification Delay-Based Strategies

To reduce alarm overload, middleware solutions introduce a delay between signal detection and alert transmission. These systems act as intermediaries, filtering alerts before sending them to clinicians’ devices. Notification delays using middleware or internal device logic filter out transient anomalies, allowing only persistent or critical events to reach staff. Studies have shown that introducing such delays can reduce false alarms [33]. Research Article [34] implemented reasoning-based middleware to defer alerts until conditions warranting escalation are met. Research Article [35] reported alarm-reduction delay strategies and alarm parameter reconfiguration. While effective in reducing alert sending frequency, this approach risks delaying true alarms, especially in rapidly deteriorating conditions. Moreover, middleware configurations require complex rule sets and maintenance, increasing system overhead. Hence, while delay-based strategies offer a practical way to reduce alarm volume, they must be complemented with real-time contextual analysis to avoid critical oversights. Overall, middleware and delay-based strategies can ease alarm overload but risk unsafe delays and require complex maintenance [36]. These highlight the demand for more adaptive, context-aware alternatives.

### 2.3. Alarm Validation Approaches

Alarm validation refers to determining the clinical or technical validity of an alarm before notifying caregivers. This is especially crucial in elderly care settings where patient variability is high, and false positives are common. Statistical process control (SPC) methods, such as CUSUM and Shewhart charts, monitor deviations from individualized baselines but often ignore autocorrelation or patient-specific thresholds [37]. Knowledge-based systems attempt to integrate clinical rules and expert knowledge for alarm interpretation [38], yet these systems are often brittle and require constant rule updates. Bayesian networks and fuzzy logic systems have shown potential for probabilistic reasoning and soft classification of alarm relevance, particularly in uncertain or imprecise clinical environments [39]. Bayesian networks allow for probabilistic validation of alarms using dependencies between physiological parameters [40]. Multivariate logic-based systems use combinations of signals and thresholds to identify alarm-relevant scenarios with greater specificity of alerts [41]. However, these methods often overlook behavioral and environmental context, such as nurse–resident distance and mobility status [20,42,43,44], patient-specific care dependency and response needs, and forecasted behavior (e.g., a slow attempt to stand up that may lead to a fall). In short, alarm validation approaches add clinical meaning to alarms. However, their lack of behavioral and environmental context restricts their effectiveness in LTC, motivating richer, context-driven methods.

### 2.4. Challenges and Motivation

Alarm management remains a persistent challenge in long-term care (LTC) facilities, where elderly individuals require nuanced, personalized attention and caregivers operate under high-workload conditions. Despite considerable efforts to address alarm fatigue in clinical settings, especially in long-term care (LTC) environments, most existing alarm management strategies remain insufficient. We identify three primary limitations across current alarm systems.

- Clinically non-interpretable suppression often leads to false negatives in LTC, where subtle transitions carry significant risk. Our proposed approach addresses this by discarding unattainable alerts by rules based on patient condition, nurse-to-patient distance, and nurse and patient speed.
- Rigid or middleware-based delays can endanger timely responses and impose technical overhead. We replace these with dynamic, context-driven delay mechanisms that balance responsiveness and workload.
- Context-unaware validation fails to capture care priorities, ignoring workload and resident-specific needs. Hence, we integrate spatial, temporal, and clinical context to ensure alerts are both technically valid and clinically actionable.

Together, these improvements form our hybrid framework integrating rule-based and LLM reasoning, designed to reduce false alarms, enhance clinical relevance, and ensure more technically valid and clinically actionable responsive care in LTC facilities.

## 3. Problem Statement

Alarm systems in LTC often generate excessive, non-specific alerts, leading to false positives, missed events, and caregiver fatigue. We propose a hybrid Context-Aware Alert System that integrates rule-based suppression, context-sensitive delays, and LLM-driven validation using clinical, spatial, and temporal features. By assigning alerts to the most suitable caregiver based on proximity, response time, and activity context, the system reduces alarm overload and ensures timely, efficient, and relevant interventions. Let the set of alarms generated over a monitoring period be denoted by $A=\{a_{1},a_{2},\ldots ,a_{N}\}$. Each alarm $a_{i}$ is represented as a tuple:

(1) $$a_{i}=\left(t_{i},x_{i},u_{i},c_{i},r_{i}\right)$$

where $t_{i}$ is the temporal context, $x_{i}$ is the spatial metric, $u_{i}$ is the urgency measure, $c_{i}$ is the clinical context, and $r_{i}$ is the resource context. The system comprises three essential decision functions used to optimize alert handling in long-term care environments:

Suppression Function ($S(a_{i}))$:

The suppression function $S\left(a_{i}\right)\in \left\{0,1\right\}$ determines whether an alarm $a_{i}$ is unreachable or non-actionable and filters it out before further processing. Suppression is based on interpretable rules derived from three dimensions:

- Distance Constraint: alarms are suppressed if the nurse is beyond a maximum reachable distance $\theta_{x}$.
- Urgency Constraint: alarms triggered by very slow or clinically insignificant motion, i.e., trying to stand up slowly or moving to a side ($u_{i}<\theta_{u}$) are suppressed.
- Care Level Constraint: if the care level $c_{i}$ of the elderly resident indicates low dependency or low fall risk, alerts are suppressed unless overridden by urgent context.

This eliminates unreachable or non-actionable alerts early in the pipeline and improves both efficiency and clinical interpretability.

Delay Function ($D(a_{i})$):

The delay function $D\left(a_{i}\right)\in R_{\geq 0}$ computes a personalized delay before the alarm is sent to caregivers. It balances system responsiveness against caregiver burden by dynamically calculating delay using:

- Urgency: faster patient movement (e.g., abrupt standing up) implies shorter delays.
- Nurse Workload: overloaded nurses should receive alerts slightly later to reduce interruptions if the event is non-urgent.
- Resident Stability: higher risk of fall or instability reduces permissible delay.

This formulation optimizes the trade-off between timely intervention and alert volume. Low-urgency alerts are delayed to avoid unnecessary interruption, while high-urgency ones are dispatched promptly.

Validation Function ($V(a_{i})$):

The validation function $V\left(a_{i}\right)\in \left\{0,1\right\}$ determines whether an alarm is clinically meaningful and should be escalated after suppression and delay. This validation is performed by computing a dynamic priority score using an LLM-based reasoning module. Due to the logical reasoning ability we opted for LLM-based validation. The target of this function is to validate only those alerts that are both technically triggered and clinically warranted. This step ensures alert escalation aligns with patient care plans and the operational context, reducing alert fatigue and enhancing caregiver focus.

Optimization Goal:

Definition of the set of effective alerts:

(2) $$A^{*}=\left\{a_{i}\in A|S\left(a_{i}\right)=1\wedge t_{i}+D\left(a_{i}\right)\leq T_{\max}\wedge V\left(a_{i}\right)=1\right\}$$

Our system aims to minimize false negatives ($\sum_{a_{i}\in A}⊮\left[y_{i}=1\wedge V\left(a_{i}\right)=0\right]$), minimize false positives ($\sum_{a_{i}\in A}⊮\left[y_{i}=0\wedge V\left(a_{i}\right)=1\right]$), minimize alert volume ($|A^{*}|$), and maximize timely coverage ($\mathrm{coverage}\left(A^{*}\right)\;\mathrm{within}\;T_{\max}$). This hybrid, context-aware nurse alerting framework leverages structured suppression logic, personalized delay, and intelligent LLM-driven validation to issue fewer but more meaningful alerts. By integrating spatial, temporal, and clinical information into the decision-making pipeline, the system improves both the precision of alarm delivery and the timeliness of caregiver intervention—addressing the pressing challenge of alarm fatigue while supporting safer elderly care.

## 4. Context-Aware Alert System

**Figure 2:**
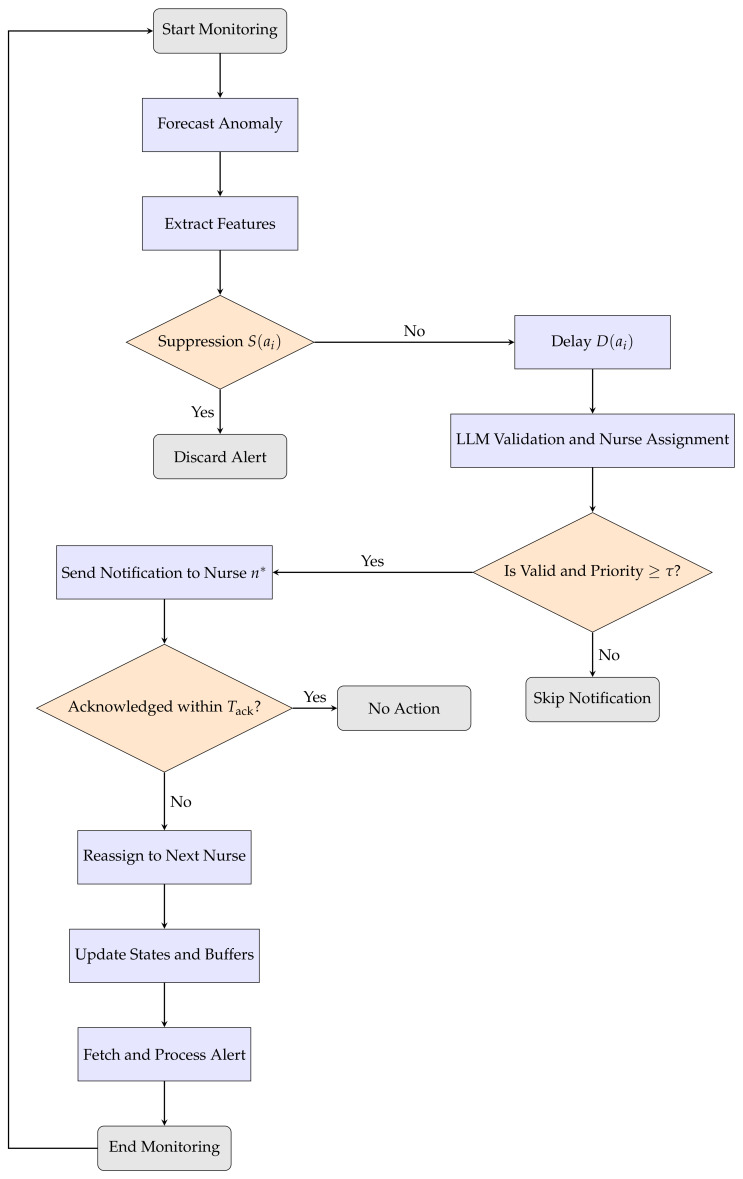
Flowchart of context-aware alerting system.

In this section, we provide a detailed explanation of the anomaly detection, suppression, and validation process, along with the notification procedure. We have used YOLOv7 custom object detection-based person identification [45] and tracking [46] to identify and track the elderly and nurses. We used YOLOv7-pose [45] to extract 2D keypoints from the video. With the help of Facebook prophet [47], anomalous activities are detected for each frame. The alert set is initialized for all the abnormal activities detected in a frame. Each alarm tuple contains information such as time, motion intensity, urgency level, care level, and nurse–resident distance. An empty alert set is initialized to store only the effective alerts. Each alarm is first checked to remove false or unnecessary ones. Alerts with low urgency, high stability, or low care priority are discarded to reduce false alarms and nurse fatigue. For the remaining alerts, a delay time is calculated using factors such as urgency, care level, and distance between nurse and resident. Alerts that would take too long to process (beyond the acceptable acknowledgment time) are discarded. Each valid alert is then evaluated using an LLM, which determines its clinical priority and recommends the most suitable nurse based on the situation. Alerts with low priority scores are ignored, ensuring that only meaningful alerts are sent. The selected nurse receives the alerts, and a timer starts to monitor acknowledgment. If no response is received within the set time limit, the alert is automatically escalated to the next available nurse. After acknowledgment, the system updates nurse workload and adds the handled alert to the effective alert set. The final output is a list of all processed and escalated alerts. The complete algorithm is presented in Algorithm 1, with key component notations summarized in Table 1. The overall system workflow is illustrated in Figure 2.
**Algorithm 1** Context-Aware Alert Decision Framework  1:**Input:** Set of alarms $A=\{a_{1},a_{2},\ldots ,a_{N}\}$, where $a_{i}=\left(t_{i},x_{i},u_{i},c_{i},r_{i}\right)$  2:**Initialize:** Effective alert set $A^{*}\leftarrow Ø$  3:**for all** $a_{i}\in A$ **do**  4:    **// Suppression Check**  5:    **if** $x_{i}>\theta_{x}$ **or** $u_{i}<\theta_{u}$ **or** $care\left(c_{i}\right)\in \left\{\mathrm{low}\text{-}\mathrm{priority}\right\}$ **then**  6:        Discard $a_{i}$ and log suppression  7:        **continue**  8:    **end if**  9:    **// Context-Aware Delay Assignment**10:    Compute delay: $D\left(a_{i}\right)=\alpha_{1}\left(1-u_{i}\right)+\alpha_{2}r_{i}+\alpha_{3}c_{i}$11:    **if** $t_{i}+D\left(a_{i}\right)>T_{\mathrm{ack}}\;\mathrm{then}$12:        Discard $a_{i}$ due to unacceptable delay13:        **continue**14:    **end if**15:    **// LLM-Based Validation and Nurse Assignment**16:    Generate prompt: $\mathrm{Prompt}\left(a_{i}\right)=T\left(t_{i},x_{i},u_{i},c_{i},r_{i}\right)$17:    Query LLM to get: $\mathrm{priority}(a_{i})$ and assigned nurse $n^{*}$18:    Compute dynamic threshold $\tau (c_{i},x_{i},r_{i},t_{i})$19:    **if** $\mathrm{priority}\left(a_{i}\right)<\tau \left(c_{i},x_{i},r_{i},t_{i}\right)$ **then**20:        Discard $a_{i}$ (Not clinically significant)21:        **continue**22:    **end if**23:    **// Notification Dispatch**24:    Send alert $a_{i}$ to nurse $n^{*}$25:    Start acknowledgment timer $T_{\mathrm{ack}}$26:    **if** No acknowledgment within $T_{\mathrm{ack}}$ **then**27:        Escalate to next-best nurse $n^{\prime }$28:    **end if**29:    Update workload and availability for assigned nurse30:    Add $a_{i}$ to effective alert set: $A^{*}\leftarrow A^{*}\cup \left\{a_{i}\right\}$31:**end for**32:**Output:** Escalated and handled alert set $A^{*}$

### 4.1. Anomalous Event Detection

Because of asynchrony in real-time monitoring, there is often a noticeable delay between observing an event and responding to it. Instead of depending only on the current motion path, it becomes crucial to predict the person’s next movement and the activity they are likely to perform. Since abnormal behaviors can result from physical or environmental factors, this study addresses the issue by forecasting elderly motion through joint trajectory prediction and activity state estimation, allowing caregivers to act proactively. In our context, abnormal behavior mainly refers to pre-fall conditions such as sudden attempts to stand up or unstable standing posture.

Let q(t) be the 3D position vector of a key joint (e.g., hip, knee, shoulder) at time *t*. The input sequence to the forecasting model is:

(3) $$X\left(t\right)=\left[q\left(t-N\right),q\left(t-N+1\right),\ldots ,q\left(t-1\right)\right]\in R^{N\times 3}$$

We use an LSTM model F to predict the joint’s future position:

(4) $$\hat{q}\left(t+\Delta t\right)=F\left(X\left(t\right)\right)$$

After the future trajectory is forecasted, this data is fed to Facebook prophet to identify abnormalities before it even occurs. Abnormal behavior is identified by examining the magnitude or pattern of these residuals. Large residuals or patterns that deviate significantly from the typical behavior of residuals are considered abnormal behavior in the data. Let y(t) be the observed value at time *t*, and $\hat{y}\left(t\right)$ be the predicted value by Prophet. The residual at time *t* is given by:

(5) $$\mathrm{Residual}\left(t\right)=y\left(t\right)-\hat{y}\left(t\right)$$

Mathematically, the abnormality score is A(t) for each time point *t* based on the residuals. It is calculated by:

(6) A(t)=|Residual(t)|

A threshold $\tau_{A}$ is used to classify behavior as abnormal:

(7) $$\mathrm{Abnormal}\;\mathrm{if}\;A\left(t\right)>\tau_{A}$$

### 4.2. Feature Extraction and Representation Module

Following the detection or forecasting of an anomalous event, the system enters the Feature Extraction and Representation Module, which plays a critical role in constructing a high-dimensional, context-rich feature representation for each alarm event. This representation is essential for the subsequent suppression, delay assignment, and validation modules that rely on clinical, spatial, and temporal information.

Given a detected or forecasted anomaly at time *t*, the system extracts multi-contextual features from real-time monitoring data, motion forecasts, care records, and nurse schedules. Each alarm $a_{i}$ is encoded as a structured tuple, $a_{i}=\left(t_{i},x_{i},u_{i},c_{i},r_{i}\right)$, as shown in Equation (1), where $t_{i}$ is the timestamp of the detected or predicted anomalous event, $x_{i}$ represents the spatial context, $u_{i}$ encodes the urgency context, $c_{i}$ captures the clinical context of the elderly, and $r_{i}$ includes resource-specific attributes. Each component of the tuple is derived using specialized extraction procedures:

(1) Temporal Context ($t_{i}$):

The temporal feature $t_{i}$ contains the timestamp of the detected or predicted anomalous event and nurse speed. The YOLOv7 pose model detects human figures in each video frame and estimates the 2D positions of keypoints (such as head, shoulders, elbows, hips, knees, and ankles). These positions are usually expressed in pixel coordinates within the frame. To compute the nurse speed of motion, we analyze how the positions of these keypoints change over time (i.e., across frames). Since video is a time-sequential medium, changes in keypoint positions can reflect movement. By measuring how much a person’s keypoints have shifted from one frame to the next and knowing the time difference between frames (based on the video frame rate), we can estimate how fast the body is moving.

Let *N* be the total number of keypoints per person, $P_{i}^{\left(t\right)}=\left(x_{i}^{\left(t\right)},y_{i}^{\left(t\right)}\right)$ be 2D coordinates of keypoint *i* at frame *t*, *f* be the frame rate in frames per second (fps), $\Delta t=\frac{1}{f}$ be the time between two frames in seconds, and *s* be the scale factor in meters per pixel. For each keypoint *i*, compute the Euclidean distance between frames *t* and t+1:

(8) $$\Delta P_{i}^{\left(t\right)}=∥P_{i}^{\left(t+1\right)}-P_{i}^{\left(t\right)}=\sqrt{{\left(x_{i}^{\left(t+1\right)}-x_{i}^{\left(t\right)}\right)}^{2}+{\left(y_{i}^{\left(t+1\right)}-y_{i}^{\left(t\right)}\right)}^{2}}$$

Convert pixel displacement to meters using the scale factor:

(9) $$P_{i,\mathrm{meters}}^{\left(t\right)}=s\cdot P_{i}^{\left(t\right)}$$

Calculate speed in meters per second:

(10) $$v_{i}^{\left(t\right)}=\frac{P_{i,\mathrm{meters}}^{\left(t\right)}}{\Delta t}=s\cdot f\cdot P_{i}^{\left(t\right)}$$

YOLOv7-pose provides 2D keypoint coordinates for each detected person per video frame. To calculate the full-body motion speed, we analyze how these keypoints move over time, convert pixel displacement into meters, and compute speed using frame rate.

(2) Spatial Context ($x_{i}$):

The spatial feature $x_{i}$ is extracted using the Euclidean distance between the elderly and nurses. The nurse to elderly distance is calculated based on the distance between the centroid of their respective bounding boxes. A bounding box in a two-dimensional image or video frame is typically represented by its top-left and bottom-right coordinates of a person or object. The centroid is the average of the bounding box’s horizontal and vertical boundaries. This gives the center point of the rectangle, which is especially useful in applications such as object detection, tracking, and activity recognition. By computing this central point, we can monitor object movement across frames or analyze motion trajectories more effectively. Let a bounding box be defined by its top-left and bottom-right corner coordinates:

(11) $$\left(x_{\min},y_{\min}\right)\;\mathrm{and}\;\left(x_{\max},y_{\max}\right)$$

The centroid $\left(x_{c},y_{c}\right)$ of a bounding box is calculated as:

(12) $$x_{c}=\frac{x_{\min}+x_{\max}}{2},\;y_{c}=\frac{y_{\min}+y_{\max}}{2}$$

Let Box A and Box B have centroids $C_{A}=\left(x_{A},y_{A}\right)$ and $C_{B}=\left(x_{B},y_{B}\right)$, respectively. Then, the Euclidean distance $x_{i}$ between the two centroids is given by:

(13) $$x_{i}=\sqrt{{\left(x_{B}-x_{A}\right)}^{2}+{\left(y_{B}-y_{A}\right)}^{2}}$$

Substituting centroid expressions for Box A and Box B:

(14) $$x_{A}=\frac{x_{\min}^{A}+x_{\max}^{A}}{2},\;y_{A}=\frac{y_{\min}^{A}+y_{\max}^{A}}{2}$$

(15) $$x_{B}=\frac{x_{\min}^{B}+x_{\max}^{B}}{2},\;y_{B}=\frac{y_{\min}^{B}+y_{\max}^{B}}{2}$$

Therefore, the Euclidean distance between two bounding boxes is:

(16) $$x_{i}=\sqrt{{\left(\frac{x_{\min}^{B}+x_{\max}^{B}}{2}-\frac{x_{\min}^{A}+x_{\max}^{A}}{2}\right)}^{2}+{\left(\frac{y_{\min}^{B}+y_{\max}^{B}}{2}-\frac{y_{\min}^{A}+y_{\max}^{A}}{2}\right)}^{2}}$$

(3) Urgency Context ($u_{i}$):

The urgency score quantifies how quickly an event may require a nurse’s intervention. It is derived from motion characteristics and historical incident risk. Let $v_{i}$ be the velocity of the elderly, $a_{i}$ be the acceleration (rate of change of movement), $s_{i}$ be the semantic activity category, and $f_{i}$ be the number of recent abnormal behavior flags. The urgency score for the elderly resident *i* is defined as:

(17) $$\mathrm{urgency}\left(u_{i}\right)=\gamma_{1}\cdot \sigma \left(v_{i}\right)+\gamma_{2}\cdot \sigma \left(a_{i}\right)+\gamma_{3}\cdot I\left(s_{i}\in R\right)+\gamma_{4}\cdot f_{i}$$

where $I(s_{i}\in R)$ is an indicator function that returns 1 if the activity $s_{i}$ belongs to the set of risky activities *R* (e.g., trying to stand up), and 0 otherwise, and $\gamma_{j}\in \left[0,1\right]$ are weighting parameters such that $\sum_{j=1}^{4}\gamma_{j}=1$. Currently, the weights are manually set based on domain knowledge. Frequent anomaly occurrence is considered the most important, $\gamma_{4}=0.35$, followed by the activity category, $\gamma_{3}=0.25$. The elderly resident’s velocity and acceleration are given equal importance, $\gamma_{1}=0.2$ and $\gamma_{2}=0.2$.

(4) Clinical Context ($c_{i}$):

The care level (CL) is extracted from resident records and encoded on a scale of 1 to 5 where lower care levels indicate higher autonomy and higher care levels suggest increased dependency. We also added scores of assistance requirements for five key activities: meals, bath, excretion, movement, and dress-up. The scores of assistance levels (ALs) are given as follows: full assistance = 5, partial assistance = 4, reminder = 3, check = 2, and independence = 1. We define a clinical priority mapping:

(18) $$\mathrm{priority}\left(c_{i}\right)=\left\{\begin{array}{cc} \mathrm{high}, & \mathrm{if}\;(\mathrm{CL}\in \{3>2>1\})\wedge (\mathrm{AL}\in \{4>3>2>1\})\wedge (\mathrm{CL}\;\mathrm{and}\;\mathrm{AL}\neq 1)\\ \mathrm{mid}, & \mathrm{if}\;\mathrm{CL}=1\wedge \mathrm{AL}=1\\ \mathrm{low}, & \mathrm{if}\;\mathrm{CL}\in \{5>4\}\wedge \mathrm{AL}=5 \end{array}\right.$$

(5) Resource Context ($r_{i}$):

The resource context is computed by tracking real-time or logged data on nurse availability. If $nurses_{active}$ is the set of on-duty nurses and task(n) indicates whether a nurse is busy, we define nurse workload as:

(19) $$r_{i}=\frac{1}{|nurses_{active}|}\sum_{n\in nurses_{active}}task\left(n\right)$$

where task(n)=1 if the nurse is currently engaged, and 0 otherwise.

(6) Feature Vector Construction:

These extracted components are then combined into a joint feature vector:

(20) $$f_{i}=\left[t_{i},x_{i},u_{i},c_{i},r_{i}\right]\in R^{d}$$

This unified vector $f_{i}$ is used for all downstream decisions, including suppression, delay adjustment, and alert validation. This module transforms raw physiological and contextual data into a structured, multi-dimensional representation that captures the clinical, environmental, and operational significance of each alarm. It forms the backbone of the decision-making pipeline in the proposed context-aware alert system.

### 4.3. Rule-Based Suppression Module

The Rule-Based Suppression Module is the first decision gate in the hybrid nurse alerting framework. Its purpose is to eliminate clinically non-actionable or physically unattainable alarms before they propagate through the system—thereby preventing unnecessary caregiver distraction, reducing system load, and minimizing the risk of alarm fatigue. This component operates based on interpretable, clinician-informed decision rules, rather than black-box logic, to ensure transparency and trust in safety-critical environments like LTC facilities. This module suppresses alarms that fall into the following categories:

- Unreachable Alarms: This condition arises when a caregiver cannot reach the elderly within a clinically acceptable time window. In our study, we define this as the nurse failing to arrive within Δt=1 s, a value chosen empirically based on the nurse movement speed in our case study. It is adjustable depending on the requirements of the deployment setup. Here, $\Delta t=t_{\mathrm{elderly}\text{-}\mathrm{stand}\text{-}\mathrm{up}\text{-}\mathrm{time}}-t_{\mathrm{nurse}\text{-}\mathrm{to}\text{-}\mathrm{elderly}\text{-}\mathrm{reaching}\text{-}\mathrm{time}}$, and the acceptable reaching time is expressed as $t_{a}=t_{\mathrm{elderly}\text{-}\mathrm{stand}\text{-}\mathrm{up}\text{-}\mathrm{time}}-\Delta t$.
- Unattainable Alarms: Alarms that are technically triggered but represent conditions that do not require further intervention. For our considered scenario, this condition is applied for fully dependent elderly residents. As there is always one attendant nurse beside them, there is no necessity for the alarm to be generated.
- Non-Urgent Context: Alarms where resident movement or posture change is within the normal behavioral profile based on their care level and history.

Thus, the suppression function is:

(21) $$S\left(a_{i}\right)=\left\{\begin{array}{cc} 0, & \mathrm{if}\;x_{i}>\theta_{x}\;(\mathrm{Too}\;\mathrm{far})\\ 0, & \mathrm{if}\;u_{i}<\theta_{u}\;(\mathrm{Not}\;\mathrm{urgent})\\ 0, & \mathrm{if}\;\mathrm{care}\left(c_{i}\right)\in \left\{\mathrm{low}\;\mathrm{priority}\right\}\;(\mathrm{fully}\;\mathrm{dependent})\\ 1, & \mathrm{otherwise} \end{array}\right.$$

where $x_{i}$ is the nurse-to-resident distance, calculated by $t_{nurse-to-elderly-reaching-time}\times v_{nurse-speed}$, $u_{i}$ is the urgency score derived from movement speed, posture risk, etc., $\theta_{x}$ is the maximum reachable distance threshold ($t_{a}\times v_{nurse-speed}$), $\theta_{u}$ is the minimum urgency threshold (e.g., motion entropy above baseline), and $\mathrm{care}(c_{i})$ is the mapping of care context priority. If $S(a_{i})=0$, the alarm is discarded and not passed to subsequent modules. If $S(a_{i})=1$, the alarm proceeds to the Delay Function and Validation Function. All suppressed alarms are stored in a shadow log for retrospective analysis and refinement.

### 4.4. Context-Aware Delay Assignment Module

The Context-Aware Delay Assignment Module is designed to mitigate alert fatigue in long-term care (LTC) environments by strategically deferring non-urgent alerts without compromising clinical safety. Unlike static delay mechanisms that treat all alerts equally, this module assigns dynamic, personalized delays based on real-time evaluation of the care environment. In LTC settings, not every alert requires an immediate response. For example, a stable patient slowly rising from a chair may not demand the same urgency as a resident with a fall risk attempting to walk unassisted. Therefore, timing becomes a key variable: this module ensures urgent alerts remain interruptive, while lower-priority alarms are queued for deferred attention based on workload, patient stability, and urgency context. The delay function $D(a_{i})$ determines how long to defer its notification:

(22) $$D\left(a_{i}\right)=\alpha_{1}\cdot \left(1-\mathrm{urgency}\left(u_{i}\right)\right)+\alpha_{2}\cdot \mathrm{resource}\left(r_{i}\right)+\alpha_{3}\cdot \mathrm{care}\left(c_{i}\right)$$

where urgency $\left(u_{i}\right)\in \left[0,1\right]$ is a normalized urgency score (higher = more critical), resource $\left(r_{i}\right)\in \left[0,1\right]$ indicates current nurse workload, and care $\left(c_{i}\right)\in \left[0,1\right]$ represents elderly physical and clinical condition-based priority (for mid priority, we choose a longer delay compared to high priority condition). Tunable coefficients $\alpha_{1},\alpha_{2},\alpha_{3}\in \left[0,1\right]$ are $\alpha_{1}+\alpha_{2}+\alpha_{3}=1$. For our case we chose these parameters from domain knowledge: $\alpha_{1}=0.5$, $\alpha_{2}=0.25$, and $\alpha_{3}=0.25$. Thus, the delay time before alert delivery is $D\left(a_{i}\right)\in R_{\geq 0}$. For high urgency, lower delay is assigned; for high nurse workload, higher delay (if safe to defer) is assigned; and for mid priority, high delay (lower intervention need) is assigned. This module ensures that care teams are not overwhelmed by alarms while safeguarding that critical interventions are never delayed beyond clinically acceptable thresholds.

### 4.5. LLM-Driven Validation and Prioritization Module

The LLM-Driven Validation and Prioritization Module serves as the final and most adaptive stage of the alert decision process. Its main purpose is to ensure that only clinically relevant and contextually important alerts are sent to the most appropriate nurse, thereby reducing false positives, filtering out unnecessary information, and maintaining confidence in the alert system. Unlike conventional validation methods based on static thresholds, this module employs LLM reasoning to analyze complex, multi-dimensional contextual data—including spatial, temporal, and clinical information—to assess both the urgency and necessity of an alert in real time. When multiple abnormal events occur simultaneously, the LLM allocates nurses intelligently based on their current availability, preventing overload on any single caregiver. The module constructs structured and interpretable prompts using these real-time contextual features to support transparent and explainable decision making. The prompt generation function is defined as:

(23) $$\mathrm{Prompt}\left(a_{i}\right)=T\left(t_{i},x_{i},u_{i},c_{i},r_{i}\right)$$

where T(·) maps structured input into a fixed natural language template. Prompt structure and mapping is shown in Table 1. This prompt is designed to be concise yet context-rich, tuned for long-term care (LTC) environments. GPT-4 has been used in our study. The LLM gives outputs if this alarm should be handled as high priority and which nurse should be given priority to send the alert. To support numerical scheduling and nurse selection, the LLM output is mapped to a scalar priority score:

(24) $$\mathrm{priority}\left(a_{i}\right)=f_{\mathrm{LLM}}\left(c_{i},x_{i},u_{i},r_{i}\right)$$

where $f_{\mathrm{LLM}}$ is a prompted or learned mapping that produces: $\mathrm{priority}\left(a_{i}\right)\in \left[0,1\right]$. The alert is escalated only if:

(25) $$\mathrm{priority}\left(a_{i}\right)\geq \tau \left(c_{i},x_{i},r_{i},t_{i}\right)$$

where τ(·) is a dynamic threshold computed by the LLM based on clinical severity, nurse-to-patient distance, nurse availability or workload, time to stand up, and time to reach the elderly resident.

### 4.6. Notification Sending and Preparation for Next Alert

Once a validated alert $a_{i}$ has been assigned to a specific nurse $n^{*}$, the system proceeds to dispatch the notification. The notification is sent to the selected nurse $n^{*}$ using a multimodal delivery mechanism optimized for responsiveness and clarity. Once the notification is dispatched, the system waits for acknowledgment within a time window $T_{\mathrm{ack}}$. If no acknowledgment is received, the system triggers escalation to reassign to the next-ranked nurse $n^{\prime }$. Upon completion of alert handling (successful response or fallback), the system resets internal buffers and performs the following:

- Releases the nurse from temporary lock if acknowledgment was successful.
- Updates the workload index $\omega_{j}$ and availability status $a_{n,j}$.
- Clears the current alert context and fetches the next candidate alert $a_{i+1}$ from the queue.
- Invokes the full validation–assignment–dispatch loop for $a_{i+1}$.

The alert analysis module processes each video frame to detect and handle multiple abnormal behaviors simultaneously. When several irregular events occur in the same frame, the system uses the LLM to assign the most suitable nurse for each situation instead of defaulting to the nearest or a single nurse. This ensures that alerts are distributed intelligently, preventing any individual nurse from being overloaded. As a result, each alert is prioritized and handled in a balanced and clinically appropriate order, maintaining both patient safety and an efficient caregiver workflow.

## 5. Experiment and Analysis

In this section, we outline the data collection process, detail the validation of our proposed method, and discuss its limitations, contributions, and future directions.

### 5.1. Data Collection

The dataset was collected from Global Care, a long-term care (LTC) facility in Japan that specializes in dementia care and operates an in-house video monitoring system. In collaboration with the facility, we accessed recorded footage capturing natural lunchtime activities of residents over seven consecutive days across three dining areas (Rooms 1, 2, and 3) for the case study. Each session lasted approximately 30–60 min, corresponding to the regular lunch period. A total of 28 elderly participants aged between 64 and 95 years were included in the study, along with four nurses who provided care support during the sessions. The elderly participants were included if they were residents of the long-term care facility, capable of performing daily activities with or without assistance, and provided informed consent (or consent was obtained from their legal guardians). Participants were excluded if they did not wish to take part in the study. The residents were categorized into three care groups based on their care level: low (1–2), medium (3), and high (4–5) [48]. Low-level residents were generally independent, requiring minimal assistance or occasional reminders for meals, hygiene, or dressing, and were able to maintain an upright posture with minor age-related stooping. Medium-level residents required partial assistance for daily activities and movement, could stand or transfer with assistive devices, and often exhibited a forward-leaning or hunched posture due to reduced core strength. High-level residents were completely dependent on caregivers, mostly immobile, and showed severe postural instability without a consistent upright posture. Among the participants 6 were in the high-care group, 8 were in the medium-care group, and 14 were in the low-care group. Seating arrangements and interactions were entirely managed by facility staff, with no intervention from researchers, ensuring that all recorded behaviors reflected natural, real-world care routines.

**Figure 3:**
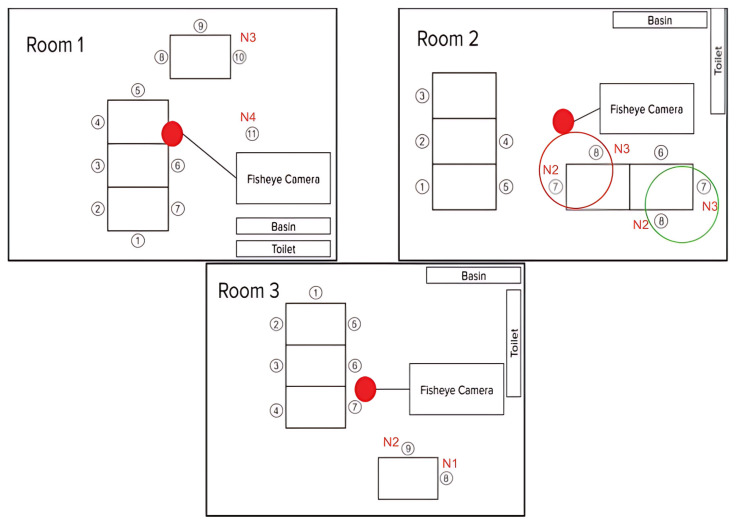
Room layout including fisheye camera placement position and sitting placement of residents. In each room, two nurses dedicatedly attended to the high care assistance required by patients by staying beside them. We also marked their positions to understand the validation later as these nurses are supposed to receive no alarm or are only alerted when there is no available option. Except for these nurses, other nurses roamed around the room to conduct various tasks. Additionally, in Room 2, residents 7 and 8 were seated in varying positions during the data collection duration so nurse 2 and 3 also changed their positions.

Importantly, no instructions were provided to either the participants or staff, ensuring that all recorded behaviors occurred naturally. For example, in Room 2, Participants 7 and 8 were seated in varying positions across sessions, reflecting routine adjustments made by caregivers based on real-time care needs. These positional changes were carefully noted during the annotation process to maintain labeling accuracy. The spatial layout of each room is depicted in Figure 3, with nurses assigned to individuals requiring higher care levels seated nearby. Their positions were also documented accordingly.

### 5.2. Validation Strategy

To rigorously assess the effectiveness of our proposed context-aware nurse alerting system, we employed a dual validation approach—spatially across rooms and temporally across days. This design allowed us to evaluate both the consistency and robustness of the system in a real-world long-term care setting. We performed a comprehensive ablation study using four alerting strategies to dissect the contribution of each component in the pipeline. The baseline method reflects the conventional ‘notify-all’ approach, where all nurses receive alerts whenever an abnormal event is detected, without regard for contextual relevance or nurse workload. This approach, while exhaustive, tends to overwhelm caregivers and generate unnecessary cognitive load. The R(S+D) strategy builds upon this by introducing rule-based suppression and delay—where alerts are blocked if contextually insignificant and delayed if premature—mimicking more practical judgment found in human care. Further extending this, the R(S+D+V) method adds a heuristic validation layer, checking the appropriateness of alerts before dispatch to enhance decision precision. Our Proposed Method combines the deterministic logic of suppression and delay with adaptive, semantic validation powered by a large language model (LLM). The rules guide initial alert filtering, while the LLM performs high-level contextual reasoning to confirm whether an alert should be sent. This hybrid approach retains interpretability while enabling flexibility in complex and ambiguous situations.

For quantitative comparison, we computed a set of performance metrics including accuracy, precision, recall, F1 score, false positives (FPs), and false negatives (FNs). These metrics were analyzed both globally and per nurse to capture variations in alert distribution, responsiveness, and error rates. Accuracy and F1 score were used to assess the overall correctness and balance between sensitivity and specificity, while FPs and FNs provided insight into the model’s reliability in suppressing false alarms and minimizing missed critical events. Tests were also conducted to see temporal (day-based) and spatial (room-based) variation.

All data were tabulated and visualized using bar charts and confusion matrices to illustrate comparative performance among the four methods. Descriptive statistics (mean and standard deviation) were used to summarize results across days and rooms, ensuring comparability between temporal and spatial dimensions. Ground truth annotations were manually created from video recordings, identifying moments when facility staff intervened by alerting nurses in response to genuine abnormal events. This human-labeled data served as the benchmark for both forecasting accuracy and alert validation, ensuring our comparisons reflected actual clinical response behavior.

The analyses were conducted using Python (version 3.9) with the NumPy, SciPy, PyCM, and scikit-learn libraries for statistical computation and metric evaluation, and Matplotlib/Seaborn for data visualization. This ensured the reproducibility, transparency, and rigorous quantitative assessment of the alert system’s performance.

### 5.3. Results

**Figure 4:**
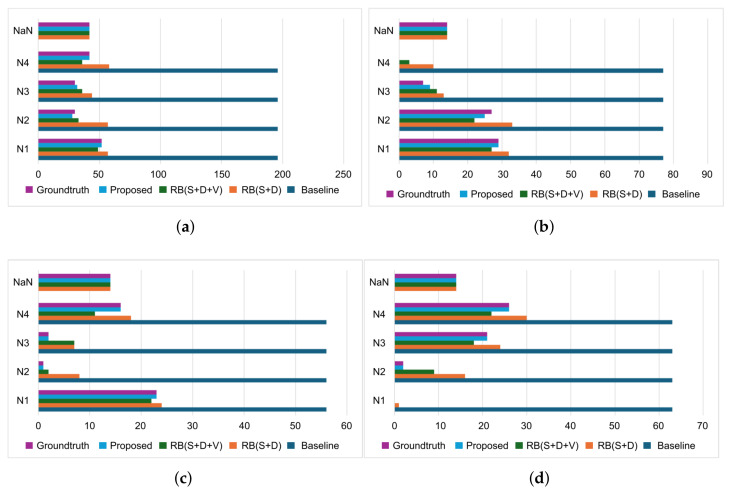
Nurse alarm load comparison for different alarm types. Here, (**a**) All = All over alarm load for each nurses for different methods, (**b**) R1 = Room 1 alarm load for each nurses for different methods, (**c**) R2 = Room 2 alarm load for each nurses for different methods and (**d**) R3 = Room 3 alarm load for each nurses for different methods. R(S+D) is rule-based suppression and delay; and R(S+D+V) adds validation layer with R(S+D). NaN = Occurrence when no alert is sent.

Figure 4 presents a comparative analysis of nurse-specific alarm loads across different alert strategies, highlighting the effectiveness of our proposed context-aware method. Compared to the baseline, which alerts all nurses indiscriminately, our model significantly reduces alert load by tailoring notifications based on situational context. In the overall case, the proposed method reduced alarm loads to 71% for Nurse 1, 71% for Nurse 2, 78% for Nurse 3, and 70% for Nurse 4. A room-wise breakdown shows consistent reductions. In Room 1, alarm loads decreased to 62% for Nurse 1, 68% for Nurse 2, 88% for Nurse 3, and, remarkably, 100% for Nurse 4. In Room 2, alert loads dropped to 59% for Nurse 1, 98% for Nurse 2, 96% for Nurse 3, and 71% for Nurse 4. In Room 3, alarm loads reduced to 100% for Nurse 1, 97% for Nurse 2, 67% for Nurse 3, and 59% for Nurse 4. These results demonstrate the model’s adaptability across spatial contexts and care configurations. On average, the proposed system achieved a 72.5% reduction in overall alert volume. When analyzing this by individual nurse, the average reduction in alerts for Nurse 1 is 73.67%, for Nurse 2 is 87.67%, for Nurse 3 is 83.67%, and for Nurse 4 is 76.67%. Notably, the model achieved complete alert suppression for Nurse 1 in Room 3 and Nurse 4 in Room 1, showing its capacity to eliminate unnecessary alerts when nurses are not contextually suited to respond. These findings underscore the model’s ability to reduce redundant notifications and distribute alerts intelligently, ultimately enhancing caregiver efficiency without compromising responsiveness.

**Figure 5:**
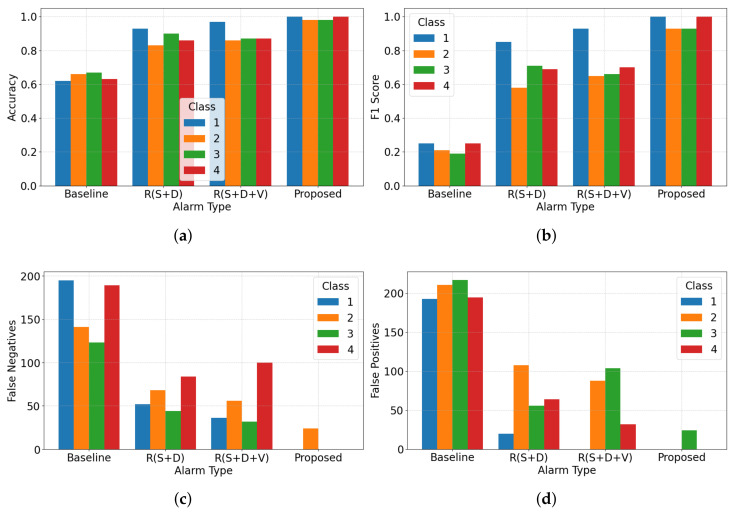
Overall performance comparison based on accuracy, F1 score, false negatives, and false positives for each nurse for different methods. R(S+D) is rule-based suppression and delay; and R(S+D+V) adds a validation layer with R(S+D). Here, (**a**) Accuracy; (**b**) F1 Score; (**c**) False Negatives and (**d**) False Positives.

**Figure 6:**
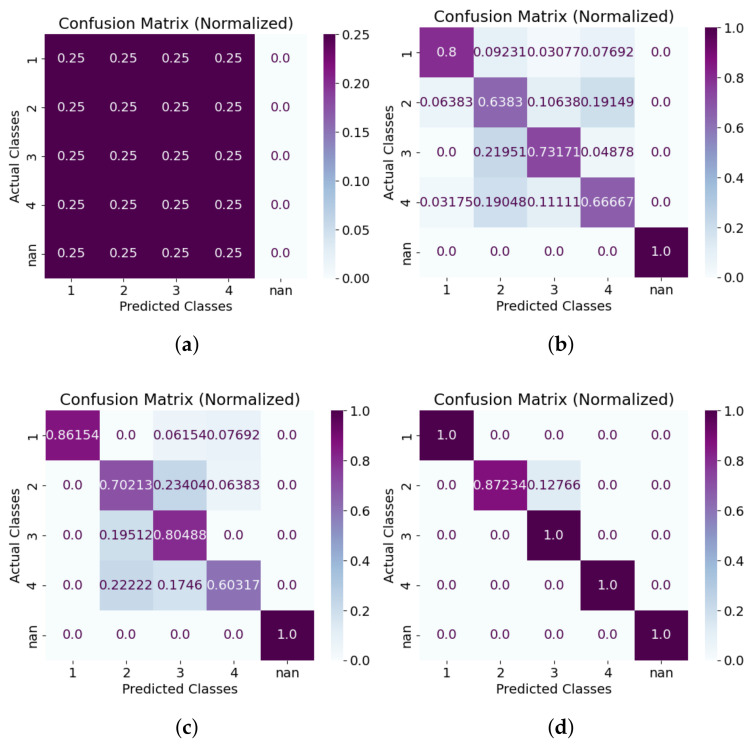
Confusion matrix for overall performance comparison for each nurse for different methods. R(S+D) is rule-based suppression and delay; and R(S+D+V) adds a validation layer with R(S+D). Here, (**a**) Baseline; (**b**) R(S+D); (**c**) R(S+D+V) and (**d**) proposed.

As illustrated in Table 2, and Figure 5 and Figure 6, the proposed context-aware alerting method consistently outperforms all other evaluated strategies across multiple evaluation metrics. The proposed model achieves a macro average F1 score of 0.97 and macro average accuracy of 0.98, underscoring its effectiveness in minimizing both false alarms and missed alerts. In terms of forecasting errors, the number of false positives (FPs) is zero for all nurses except Nurse 2, and false negatives (FNs) are zero for all nurses except Nurse 3, reflecting high precision and recall for most responders.

**Figure 7:**
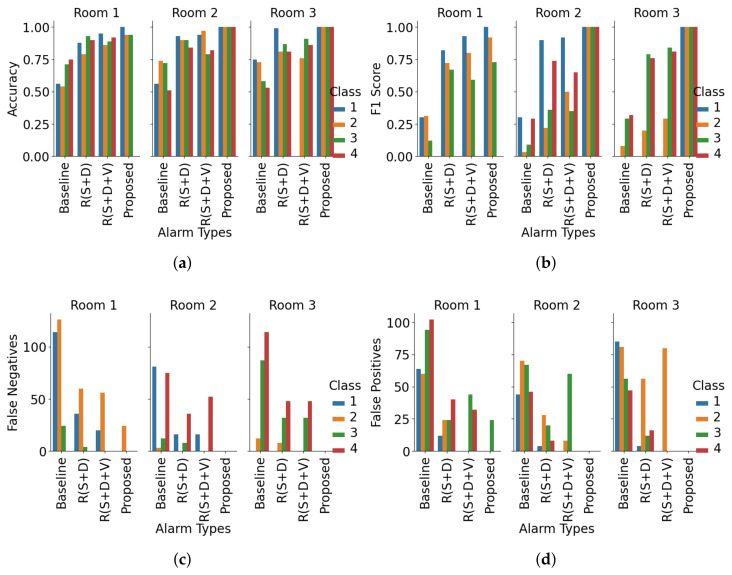
Room-wise performance comparison using accuracy (**a**), F1 score (**b**), false negatives (**c**), and false positives (**d**) as metrics. Each figure presents comparative results across three rooms and four nurses, highlighting performance variations influenced by spatial context. R(S+D) denotes rule-based suppression and delay, while R(S+D+V) represents the same with an added validation layer.

**Figure 8:**
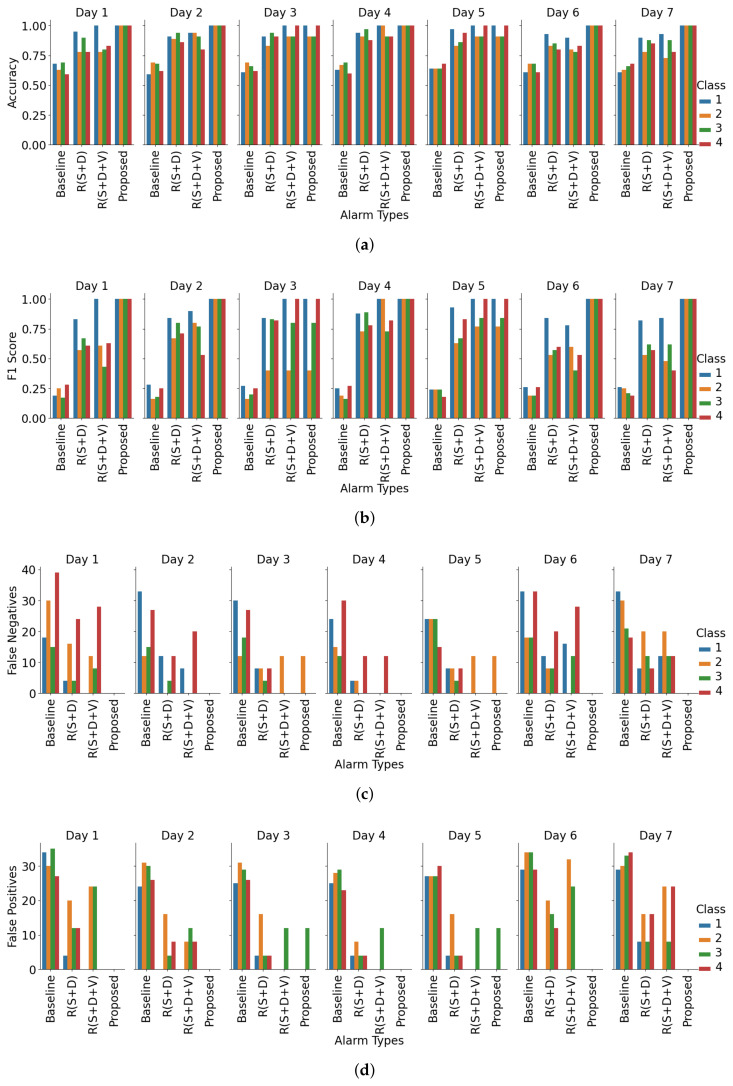
Day-wise performance comparison using accuracy (**a**), F1 score (**b**), false negatives (**c**), and false positives (**d**) as metrics. Each figure presents comparative results across three rooms and four nurses, highlighting performance variations influenced by temporal context. R(S+D) denotes rule-based suppression and delay, while R(S+D+V) represents the same with an added validation layer.

From the room-based validation in Figure 7, the proposed context-aware alerting system consistently outperforms all other strategies across multiple metrics. It achieves a macro average F1 score of 0.94 and accuracy of 0.98, effectively minimizing both false alarms and missed alerts. Accuracy across Rooms 1, 2, and 3 is 0.97, 1.00, and 0.97, while F1 scores are 0.91, 1.00, and 0.91, reflecting stable classification performance regardless of room context. Compared to day-based validation, accuracy variation is higher here, with a standard deviation of 1.73. Forecasting errors show zero false positives (FPs) except for Nurse 2 in Room 1, and zero false negatives (FNs) except for Nurse 3 in Room 1. Temporal validation over seven days (Figure 8) further confirms robustness, with the proposed method again outperforming all baselines. It achieves a macro average F1 score of 0.97 and accuracy of 0.99. Day-wise accuracy values are 1.00, 1.00, 0.97, 1.00, 0.97, 1.00, and 1.00, while F1 scores are 1.00, 1.00, 0.84, 1.00, 0.92, 1.00, and 1.00, demonstrating adaptability across operational conditions. F1 variation is higher here, with a standard deviation of 6.29. Forecasting errors show zero FNs except for Nurse 2 on Days 3 and 5, and zero FPs except for Nurse 3 on the same days. Overall, these results provide strong empirical evidence that the proposed hybrid system not only reduces unnecessary alarms but also ensures high reliability in alert recognition and nurse assignment, both spatially across rooms and temporally across daily sessions.

## 6. Discussion

This study investigated the design and evaluation of a hybrid, context-aware alerting framework for elderly care in long-term care (LTC) settings. The framework integrates deterministic rule-based suppression with flexible LLM-driven reasoning, enabling context-sensitive, clinically meaningful alerts. The proposed context-aware alerting framework demonstrated significant improvements in both alert accuracy and reduction of unnecessary notifications. Evaluated using accuracy, precision, recall, F1 score, false positives (FPs), and false negatives (FNs), the model consistently outperformed baseline and rule-based methods. Ground truth was meticulously annotated from real staff interventions in video recordings, ensuring clinical relevance in validation.

### 6.1. Contributions

In the evaluation we could validate our initial research questions from the quantitative analysis. To answer research question ‘R1’ from Figure 4, we can see that our method reduced overall nurse alarm loads to 71% (Nurse 1), 71% (Nurse 2), 78% (Nurse 3), and 70% (Nurse 4) compared to the baseline. Room-wise reductions show even greater adaptability: in Room 1, Nurse 4 experienced 100% alert suppression; in Room 3, Nurse 1’s alerts were fully suppressed. These targeted reductions reduce the 100% alarm load (baseline) to 27.5% (proposed), achieving a 72.5% reduction on average. From Table 2, and Figure 5 and Figure 6, the model achieved a macro average F1 score of 0.97 and accuracy of 0.98. Room-wise validation (Figure 7) further showed accuracies of 0.97, 1.00, and 0.97, and F1 scores of 0.91, 1.00, and 0.91 for Rooms 1, 2, and 3, respectively. Day-wise performance (Figure 8) was similarly robust: macro accuracy was ≥0.97 for all seven days, and F1 scores were ≥0.92 (except Day 3: 0.84). The overall accuracy of 0.98 and a macro F1 score of 0.97 far surpass the baseline accuracy of 0.21 and F1 score of 0.18. These results confirm that hybrid contextual alerting can effectively address alarm fatigue without sacrificing safety.

To answer research question ‘R2’ from Figure 4, we can see that compared to the baseline error rates (FPR 0.20, FNR 0.79), the method achieved drastically lower values (FPR 0.005, FNR 0.023). From Figure 7 we can see that FNs are zero except for Nurse 2 in Room 1, and FPs are zero except for Nurse 3 in Room 1, and from Figure 8 we can see that FNs are zero except for Nurse 2 on Days 3 and 5, and FPs are zero except for Nurse 3 on the same days. This confirms the utility of embedding interpretable, clinically grounded features for improving alert reliability.

To answer research question ‘R3’ from Figure 4, we can see that our proposed method achieved an accuracy of 0.98 and macro F1 score of 0.97, compared to the rule-based method (accuracy 0.78, F1 score 0.79). This validates the effectiveness of context-driven validation strategies in meeting the dynamic requirements of elderly care.

These results illustrate the clear benefits of contextual alerting in long-term care facilities. Despite the success, several areas remain for enhancement. The current model does not yet incorporate fatigue, emotional rapport, task load history, and in-depth temporal information, which significantly influence caregiver performance and trust. The lack of temporal information also reflects in the results as the day-based validation standard deviation of the F1 score is 6.29, which is higher compared to the room-based validation standard deviation of the F1 score (5.20). This indicates poorer performance in temporal variation. Caregiver specialization and dynamic task prioritization also warrant inclusion for broader applicability. Real-world deployment would require integration with feedback mechanisms, override options, and facility-specific routines. To that end, future work should focus on: embedding continuous learning from caregiver feedback, modeling longitudinal performance trends, and enabling interactive, bidirectional AI–human communication.

By combining spatial metrics with LLM-based reasoning, the system surpasses rigid rule-based filtering, enabling personalized nurse–patient matching, reducing alarm fatigue, and improving responsiveness. The hybrid approach balances interpretability and adaptability, making it well-suited for real-world elderly care, where it enhances alert efficiency, precision, and scalability.

### 6.2. Limitations and Future Challenges

While the proposed system demonstrates promising results for context-aware elderly monitoring and alerting in long-term care (LTC) environments, several limitations remain that open avenues for future improvement and practical enhancement. First, the current framework depends on prior patient information, such as care level assessment and average movement speed, to ensure accurate context modeling and alert generation. These parameters are essential for personalized monitoring, yet they pose challenges when applied to new residents without existing care history. To address this, future iterations of the system can integrate adaptive initialization modules that automatically estimate baseline parameters through short-term observation or unsupervised clustering of motion patterns during the first few days of monitoring. Such self-calibrating models would allow the system to dynamically personalize its thresholds and alerts for new patients without manual input. Second, the current study is confined to daytime group activity sessions, particularly during mealtimes, where multiple residents and nurses interact. Consequently, nighttime monitoring scenarios—such as sleeping posture analysis, nocturnal falls, or nighttime wandering—were beyond the present scope. However, because the proposed model operates on skeleton-based data, which are inherently privacy preserving, it can be extended for 24 h monitoring without compromising resident privacy. Future research could integrate low-light skeleton extraction and lightweight temporal models to extend system functionality to nighttime environments while maintaining operational continuity. Third, although broader challenges such as data privacy, budget constraints, and regulatory compliance remain important considerations, the proposed method already addresses some of these concerns by utilizing the existing video monitoring infrastructure of the facility without hardware modification. The only additional requirement is a GPU-enabled PC for processing, making deployment cost-effective and minimally intrusive. Additionally, the data utilized is skeleton data and the elderly and nurses are given a unique id for identification instead of their name, which to some extent mitigates the privacy issue. For future scalability, edge-computing integration or model optimization (e.g., pruning or quantization) could further reduce hardware dependency and energy consumption, enhancing accessibility for smaller care facilities. In summary, future extensions of this work will focus on adaptive parameter estimation, round-the-clock deployment, and resource-efficient scaling, ensuring that the system evolves into a fully autonomous, privacy-preserving, and sustainable solution for real-world elderly care environments.

## 7. Conclusions

This study presents a hybrid, context-aware nurse alerting framework for long-term care settings, aimed at mitigating alarm fatigue, caregiver overload, and delayed response in elderly monitoring. By combining rule-based filtering and delay mechanisms with semantic reasoning, the system achieved significant improvements in both efficiency and reliability. Evaluations using real-world video data demonstrated a 72.5% average reduction in nurse alert load and nearly complete elimination of false alarms, with macro F1 and accuracy scores of 0.97 and 0.98, respectively. These results validate the effectiveness of the interpretable suppression, adaptive delay, and contextual validation mechanisms proposed in this study. The framework offers a scalable path toward personalized and real-time alerting that enhances both resident safety and caregiver well-being. Future work will extend this approach to incorporate caregiver fatigue, specialization, and interactive AI–human collaboration to further advance human-centered elderly care.

## Figures and Tables

**Table 1:** Key components of the context-aware alert system.

Components	Mathematical Expressions
Time in an instant	$t_{i}$
Nurse-to-patient distance	$x_{i}$
Urgency context	$u_{i}$
Clinical context	$c_{i}$
Nurse availability	$r_{i}$
Alarm instance	$a_{i}$
Maximum reachable distance threshold	$\theta_{x}$
Minimum urgency threshold	$\theta_{u}$
Suppression component	$S(a_{i})$
Delay component	$D(a_{i})$
Dynamic threshold computed by the LLM	τ
Nurse alert acknowledgment time window	$T_{a}ck$

**Table 2:** Overall performance comparison. Here, the class represents alarms sent to each nurse. Nan represents the situation when no alarm is sent. R(S+D) is rule-based suppression and delay; and R(S+D+V) adds a validation layer with R(S+D).

Alarm Type	Class	Precision	Recall	F1 Score
Baseline	1	0.25	0.25	0.25
	2	0.18	0.25	0.21
	3	0.16	0.25	0.19
	4	0.24	0.25	0.25
	NaN	0.00	0.00	0.00
R(S+D)	1	0.91	0.80	0.85
	2	0.53	0.64	0.58
	3	0.68	0.73	0.71
	4	0.72	0.67	0.69
	NaN	1.00	1.00	1.00
R(S+D+V)	1	1.00	0.86	0.93
	2	0.60	0.70	0.65
	3	0.56	0.80	0.66
	4	0.83	0.60	0.70
	NaN	1.00	1.00	1.00
Proposed	1	1.00	1.00	1.00
	2	1.00	0.87	0.93
	3	0.87	1.00	0.93
	4	1.00	1.00	1.00
	NaN	1.00	1.00	1.00

## Data Availability

The data and source code will be made available upon request.
